# Association between Handgrip Strength and Cognitive Function in Older Adults: Korean Longitudinal Study of Aging (2006–2018)

**DOI:** 10.3390/ijerph19031048

**Published:** 2022-01-18

**Authors:** San Lee, Jae Won Oh, Nak-Hoon Son, Woojin Chung

**Affiliations:** 1 Department of Health Policy and Management, Graduate School of Public Health, Yonsei University, Seoul 03722, Korea; sanlee@yonsei.ac.kr; 2 Department of Psychiatry, Institute of Behavioral Science in Medicine, Yonsei University College of Medicine, Seoul 03722, Korea; 3 Department of Psychiatry, Yongin Severance Hospital, Yonsei University College of Medicine, Yongin 16995, Korea; jaewonoh@yuhs.ac; 4 Division of Biostatistics, Yongin Severance Hospital, Yonsei University College of Medicine, Yongin 16995, Korea; nhson@yuhs.ac; 5 Institute of Health Services Research, Yonsei University, Seoul 03722, Korea

**Keywords:** cognitive decline, handgrip strength, aging, physical strength, KLoSA, South Korea

## Abstract

Accumulating research indicates that handgrip strength is associated with cognitive function. Studies have also shown the difference in cognitive decline between males and females. We investigated the association between baseline handgrip strength and later cognitive function in older adults according to sex using the dataset from Korean Longitudinal Study of Aging (2006–2018). Overall, 9707 observations of 1750 participants (989 males and 761 females) over 65 years of age were sampled from the first wave, followed by six consecutive waves. The Korean version of the Mini-Mental State Examination and baseline handgrip strength scores were assessed. Sociodemographic and health-related variables were also included as covariates in the multivariable linear mixed models. Males in the lowest quartile of the baseline handgrip strength decreased in cognitive function (β = −0.54, standard error (SE) = 0.16, *p* < 0.001), compared to males in the highest quartile. For females, those in the second lowest quartile (β = −0.65, SE = 0.19, *p* < 0.001) and the lowest quartile (β = −0.53, SE = 0.19, *p*< 0.01) decreased in cognitive function. Handgrip strength may be positively associated with later cognitive function, but the association may be non-linear and differ between sexes. Sex-specific preventive assessment of handgrip strength may help identify older adults at risk for cognitive impairment.

## 1. Introduction

Cognitive decline and dementia are significant sources of disability among older adults [1]. The impact of aging on cognition is a public health concern, with an increasing number of studies establishing the determinants of cognitive decline and evaluating treatment strategies [2,3]. According to the World Health Organization (WHO), the number of dementia patients worldwide was 47.4 million in 2015, and it is estimated to reach 75.6 million in 2030 and 135.4 million in 2050 [4]. The 2009 World Alzheimer’s Report noted that the prevalence of dementia in East Asia is growing at a faster rate than expected, with the South Korean population continuing to age at a rate faster than people from other countries of the Organisation for Economic Co-operation and Development (OECD) [5]. Among OECD countries, South Korea (hereafter Korea) has the highest number of nursing home beds per 1000 for those aged 65 years or above [6]. Furthermore, dementia patients in Korea are expected to exceed 1 million (10.3% of older adults) by 2024 and 2 million (12.3% of older adults) by 2041, thereby increasing the socioeconomic disease burden [7].

In addition to the deterioration of cognitive function, age-related decreases in physical function represent a severe personal and social issue. Studies have reported that handgrip strength affects quality of life in older adults, with midlife handgrip strength being a major predictor of functional limitations and disability in older age [8]. Handgrip strength can be used as an index of frailty [9] and has been associated with mortality rates among older adults [10].

Accumulating evidence highlights the association between handgrip strength and cognitive function in older adults. In one prospective cohort study in the United States of America, data over a 7-year follow-up period revealed a positive association between handgrip strength and cognitive function in older adults [11]. Another longitudinal study of 877 older adults in the United States of America reported that a one-pound decrease in baseline handgrip strength indicated a subsequent 1.5% increase in the likelihood of Alzheimer’s disease [12]. Other studies have similarly reported a positive association between baseline handgrip strength and cognitive assessments [13,14].

In Korea, the Korean Longitudinal Study of Aging (KLoSA) conducted a longitudinal assessment of middle-aged and older adults and demonstrated that individuals with low handgrip strength exhibit a hazard ratio of 1.36 for cognitive impairment when compared to those with higher handgrip strength [15]. Another study also reported a bidirectional association between handgrip strength and cognitive function using the KLoSA data [16]. Although studies have investigated cognitive deterioration and handgrip strength among the aging population, a limited number of studies have evaluated this association based on sex within the Korean population.

Research has highlighted the differences in cognitive decline between males and females. Cognitive impairment and the prevalence of Alzheimer’s disease are reported to be higher in females than in males [17,18]. A recent review also emphasized the influence of sex on the prevalence and incidence of dementia [19]. Moreover, researchers have suggested that sex is a crucial variable in the heterogeneity of Alzheimer’s disease, influencing factors such as symptomatology, progression, and risk profiles [20]. Moreover, previous studies have reported that handgrip strength is higher in males than in females [21,22]. Based on the above results, studies utilizing KLoSA data have attempted to examine the temporal relationship between handgrip strength [23] and cognitive function [15] with repeated measures over time [14,24]. However, these studies did not control for the possibility that handgrip strength and cognitive function decrease simultaneously over time.

This study aimed to address the gaps identified above. We investigated the association between baseline handgrip strength and later cognitive function according to sex using a longitudinal dataset of older Korean adults. The baseline handgrip strength measurement was utilized to predict the risk of later cognitive impairment, controlling for the potential decrease in handgrip strength over time.

## 2. Methods

### 2.1. Survey Overview and Study Population

We analyzed 12 years of KLoSA data (2006–2018). The KLoSA is a biennial survey of nationally representative Koreans over the age of 45, and participants were recruited via multistage, stratified probability sampling. A total of 10,254 participants completed the baseline survey in 2006. A more detailed description of the survey can be found elsewhere [25].

**Figure 1:**
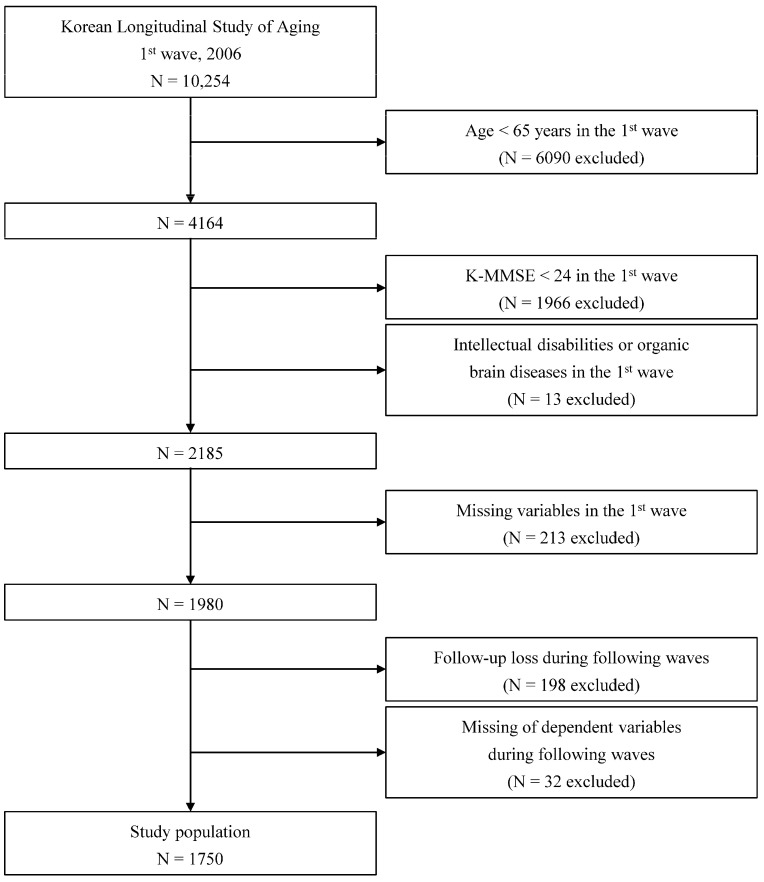
Process for selecting the study population.

For this study, we included a subset of participants aged over 65 years at the time of the baseline survey in 2006. We excluded participants with a baseline Korean mini-mental state examination (K-MMSE) score less than 24, those with intellectual disabilities, and those with organic brain diseases. Participants with missing data in the first wave (2006) or missing K-MMSE scores during the surveys were also excluded. In addition, we restricted our analysis to participants for whom cognitive function data were available on at least two occasions during the follow-up period. Consequently, 9707 observations from 1750 participants were included in the analysis at baseline. The detailed process for selecting the study population is shown in Figure 1.

The Institutional Review Board of Severance Hospital (Y-2018-0138) waived the requirement for approval and consent since the analyses of the present study were based on de-identified publicly available secondary data (http://survey.keis.or.kr, accessed on 10 November 2021). Informed consent was obtained from all participants when the KLoSA was conducted in accordance with the ethical principles of the Declaration of Helsinki. 

### 2.2. Assessment of Cognitive Function

Cognitive function was assessed using the K-MMSE [26]. The K-MMSE consists of 11 items related to orientation for time and place, memory registration and recall, attention/calculation, language, and visual construction. The total K-MMSE score ranges from 0 to 30, with cognitive impairment defined as a score of less than 24. Participants with cognitive impairment in the first wave of the survey were excluded from the analysis.

### 2.3. Assessment of Handgrip Strength

Handgrip strength was measured in kilograms using a handheld dynamometer (Tanita 6103; Tanita Corp., Tokyo, Japan). The test was performed in a sitting position with the elbow flexed to 90 degrees, and participants were instructed to squeeze the handle of the dynamometer as hard as possible. Handgrip strength was measured twice in both the left and right hand. The baseline handgrip strength of each participant was calculated as the average of all four measurements in the first wave of the survey. The mean scores were then divided into quartiles, ranging from handgrip 1 (top 25% of handgrip strength) to handgrip 4 (lower 25% of strength). The effects of sex on handgrip strength were also considered; therefore, the quartiles were separately assessed for males and females. Such a distribution of participants’ scores into quartiles followed the method of previous studies investigating the effects of handgrip strength [27]. This allows the participants’ scores to be objectively distributed into categories, which can then be compared, in this case, with cognitive function.

### 2.4. Assessment of Covariates

The following sociodemographic variables were included as potential confounders: sex, age, survey year (wave), educational attainment, economic activity, equalized household income, marital status, and residential area. The following health-related variables were also included: alcohol consumption, smoking, physical activity, chronic diseases, depressive symptoms, and body mass index (BMI). The presence of chronic diseases, including hypertension, diabetes, stroke, angina, myocardial infarction, chronic pulmonary diseases, and any type of cancer, were determined based on self-reported diagnosis by a physician. The total number of comorbid diseases was categorized as “zero,” “one,” and “two or more.” Scores of 4 or more on the 10-item short form of the Center for Epidemiologic Studies Depression Scale (CES-D10) are considered indicative of depressive symptoms [28,29]. BMI was divided into four groups based on the revised Asia-Pacific BMI criteria by the WHO Western Pacific Region [30].

### 2.5. Statistical Analysis

Baseline characteristics, including sociodemographic and health-related variables, were analyzed as frequencies and percentages, unless otherwise stated. Weight-adjusted chi-squared tests and *t*-tests were performed to evaluate differences based on sex. Multivariable analysis was performed using a linear mixed model for males and females separately. Considering the effects of sex on cognitive function and handgrip strength, the assessments were performed independently.

All variables were analyzed as time-dependent covariates (potential to change as time progressed), except for age and educational attainment. The age variable was assessed at the first wave of the survey and set as a time-fixed variable as the age would just increase concurrently with the year of the follow-up measure. The unadjusted models evaluated the association between handgrip strength and cognitive function, while the adjusted model included the covariates in the analysis, including educational attainment, economic activity, household income, marital status, residential area, and other health-related variables.

The Bayesian information criterion (BIC) was used to test the fitness of each model. Among the covariance structures, the unstructured component was associated with the lowest BIC and was therefore selected for multivariable analysis. The variance inflation factor ranged from 1.07 to 3.33 for males and from 1.03 to 3.45 for females, indicating no significant multicollinearity between the variables in any model. The regression coefficient was used to evaluate the association between cognitive function and each variable. All statistical analyses were performed using SAS (version 9.4; SAS Institute, Cary, NC, USA). The level of statistical significance was set at *p* < 0.05 (two-tailed).

## 3. Results

### 3.1. Baseline Characteristics

Table 1 shows the distribution of K-MMSE scores and handgrip strength according to sex during the first wave of the survey. The handgrip strength was 29.38 ± 0.18 kg for males and 18.09 ± 0.15 kg for females. The K-MMSE scores were 27.49 ± 0.06 in males and 26.87 ± 0.07 in females. Weight-adjusted *t*-tests revealed significant differences in K-MMSE scores and handgrip strength according to sex (all *p* < 0.001).

The baseline characteristics of the study participants according to sex are presented in Table 2. Among the 1750 participants, 989 were males and 761 were females. In addition to handgrip strength and age, all other sociodemographic characteristics of the study participants demonstrated significant differences. A greater number of males had higher educational attainment than their female counterparts, besides those with elementary or lower education. This trend was consistent with economic activity, where a significantly greater proportion of males were employed compared to females (79.0% vs. 21.0%); however, the unemployed had a similar distribution (49.1% vs. 50.9%). More males reported being married than their female counterparts (68.3% vs. 31.7%), which was consistent with the unmarried category, having a greater proportion among females in the unmarried group.

Regarding health-related variables, more females reported depression than males (57.8% vs. 42.2%). However, for alcohol consumption, smoking, and physical activity, regardless of the options within each variable, there was a higher percentage of males in all groups.

### 3.2. Cognitive Function

**Figure 2:**
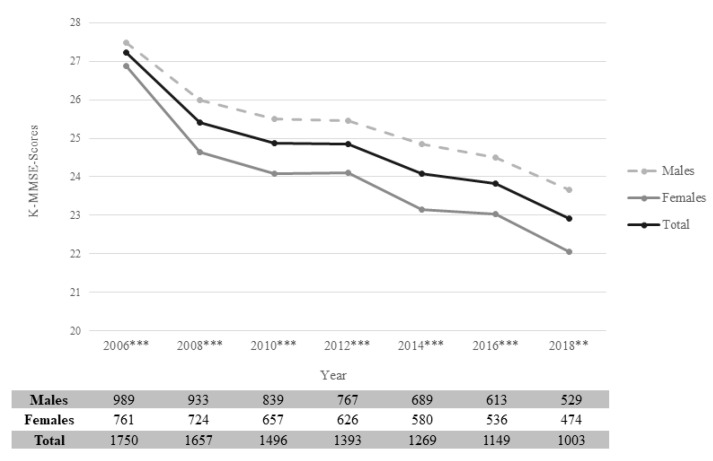
Changes in mean K-MMSE scores and the number of study participants according to sex during the seven waves of the KLoSA. K-MMSE: Korean Mini-Mental State Examination; KLoSA: Korean Longitudinal Study of Aging. ** *p* < 0.01, *** *p* < 0.001.

Figure 2 shows the mean K-MMSE scores according to sex during each survey wave. The number of study participants decreased as the survey was repeated. In the seventh wave of the survey, a total of 1003 participants were assessed. K-MMSE scores decreased over time in both sexes, although the extent of decrease differed according to sex. The K-MMSE score significantly differed according to sex in all waves of the survey (*p* < 0.001, first to sixth wave; *p* < 0.01, seventh wave). Additionally, Supplementary Figures S1 and S2 show the K-MMSE distribution according to the baseline handgrip strength group quartiles for males and females separately.

### 3.3. Association between Covariates and Cognitive Function

Table 3 shows the factors associated with the K-MMSE scores. As per the unadjusted analysis, for males, the cognitive function significantly decreased in all handgrip strength groups compared to the reference, handgrip strength group 1. For females, the unadjusted analysis reported a reduction in the lower 50th percentile of handgrip strength in groups 3 and 4 only, compared to the reference group.

When adjusting for all other covariates, males showed a reduction in cognitive function for those in the weakest handgrip strength group 4 only (β = −0.54, SE = 0.16, *p* < 0.001), while for females, there were significant reductions for those in groups 3 (β = −0.65, SE = 0.19, *p* < 0.001) and 4 (β = −0.53, SE = 0.19, *p* < 0.01), consistent with the unadjusted analysis. Therefore, sex differences were present, and the association between the baseline handgrip strength groups and cognitive decline were not linear across all handgrip strength groups. Regarding age and cognitive function, both sexes reported a reduction only for those ≥ 80 years old than in those in the 65–69 age group at baseline. 

Regardless of sex, cognitive function significantly decreased in each wave following the first wave of the survey (all *p* < 0.001), whereas educational attainment demonstrated an increase in cognitive function for both sexes compared to the elementary school reference. In addition, both males and females had reduced cognitive function for those living in rural areas compared to those living in urban areas. Both males and females reported a significant reduction in cognitive function for those with depression; however, an increase in cognitive function was reported among those who were physically active.

## 4. Discussion

In the present study, which was based on a longitudinal analysis of data collected from 2006 to 2018 in Korea, we observed a significant association between baseline handgrip strength and cognitive function in older adults. Our adjusted models showed that handgrip strength was associated with the K-MMSE score, where those in the lower baseline handgrip groups demonstrated a reduction in cognitive function at follow-up assessments for both males and females. Older females in the lower 50th percentile for handgrip strength at baseline were more likely to have a reduction in cognitive function in later years, whereas for males, such a reduction in cognitive function applied to those in the lowest 25th percentile for handgrip strength only. Hence, the results showed that there were differences among the sexes, and the association between handgrip strength and cognitive function was non-linear. Our results demonstrated that not all groups of baseline handgrip strength had a significant association regarding cognitive decline, and such differences were dependent on the sex of older adults.

The significant association between handgrip strength and cognitive function appears to be in line with previous studies [11,31]. A recent Korean study examined the association between K-MMSE scores and handgrip strength using 10 years of KLoSA data [24]. Unlike our study, the Korean study assessed adults aged over 45 years and categorized handgrip strength into five baseline groups. We assessed handgrip strength at 65 years of age instead, as we determined that 45 years may not be old enough to detect the long-term consequences of the difference in strength. Nonetheless, on average, the K-MMSE scores were 0.12 points lower in the lowest grip strength group than in the highest grip strength group. Another study conducted in China grouped the baseline group strength into quartiles and investigated the follow-up scores of episodic memory and global cognitive function. The study found that the highest quartile of grip strength was associated with better cognition and slower rates of decline [32]. Our results were consistent with those of the aforementioned studies, which showed that the baseline handgrip strength was positively associated with cognitive function. The only difference was that our study identified that the reduction in cognitive function differed according to sex. This indicates that the individuals’ baseline handgrip strength may be a factor in predicting later cognitive decline. Indeed, a recent scoping review also suggested that the relationship between handgrip strength and cognitive function is not only associative but also predictive [31].

Physiological factors may explain the association between handgrip strength and cognitive function. First, oxidative stress may mediate the association between these two factors. Previous studies have revealed that oxidative stress is not only associated with cognitive impairment [33] but also with decreases in muscle mass (i.e., sarcopenia) [34]. Second, since cognitive function and muscle strength are both related to executive function, entities affecting the nervous system, such as chronic inflammation, may be associated with changes in cognitive function [35] as well as sarcopenia [36]. Additional research has suggested that damage to the cerebral vasculature or degenerative changes in the brain can affect brain regions responsible for cognition and movement [37]. In addition, cognitive impairment may lead to decreased physical activity, thereby leading to decreased muscle mass and grip strength [16]. Given these potential mechanisms, lifestyle factors, such as physical activity, may play a role in improving neuronal plasticity and cognitive function and in preventing the deterioration of grip strength [38].

The effects of sex differences on cognitive decline were also demonstrated. Although slightly different from the unadjusted analysis, when considering all sociodemographic and health factors, females reported cognitive impairment for those in the lower 50th percentile, whereas it was only the lowest 25th percentile group for males. Prior studies have reported that females had faster cognitive deterioration compared to males across various cognitive domains, including visual and verbal processing, as well as semantic and episodic memory [18]. Such differences between males and females have been confirmed from early developmental stages to later life [17]. Additional clinical studies have also shown that females carry a heightened risk of developing Alzheimer’s disease pathology compared to males after controlling for age, thus supporting the sex differences reported in the current study [39]. Therefore, along with the prior research, which showed females experiencing cognitive decline in larger percentile compared to males, the findings of the current study also suggest that females may be at a greater risk of cognitive decline if they have lower handgrip strength in earlier stages of life.

From a cultural standpoint, the differences between men and women were in accordance with other East Asian countries where gender roles have remained static in older age groups. Studies have reported that men are typically more engaged in activities that accumulate intellectual experiences through education and occupation than women [40,41,42]. Therefore, factors such as age, education, and social activities have stimulated the gender effect in cognitive function and decline. However, such differences in cognitive function were diminished in a more recent study controlling for education, possibly arguing for the fact that the more equally educated generation may have less disparity in cognitive function and decline [43]. Further research into the causation between education and cognitive function should be conducted with a more diverse sample.

Cognitive function was associated with other sociodemographic factors. We assessed age as a time-fixed variable from the first wave of the survey to avoid multicollinearity between the survey year and age group variables. The results demonstrated that the survey year reflects the cohort effect and the passage of time. Physical activity and depressive symptoms were also significantly associated with cognitive function in both sexes. The association between physical activity and cognitive function has been reported in previous studies [44,45]. Likewise, studies have also suggested that depression is a risk factor for dementia [46,47], and our results indicate that depressive symptoms are related to low cognitive function. Furthermore, cognitive function was significantly higher among those employed and lower in participants with two or more chronic diseases than in those without chronic diseases for males only. This corresponded with the previous literature, which already suggested several comorbid diseases such as hypertension [48], type 2 diabetes [49], chronic obstructive pulmonary disease [50], and any cancer or cancer-related treatments [51] as risk factors for Alzheimer’s disease, vascular dementia, and cognitive impairment. Furthermore, underweight females had significantly reduced cognitive function, whereas this effect was absent in males. Similar findings were identified, with underweight being a risk factor for cognitive decline among females, whereas elevated BMI was a risk factor for males [52,53].

In this study, baseline handgrip strength was a factor for predicting the risk of later cognitive impairment, which took into account the continued long-term influence of handgrip strength on cognitive function. However, this study had some limitations. First, we were unable to identify a causal relationship between handgrip strength and cognitive function. Second, we could not include some potential confounding factors, such as intelligence quotient, family history of dementia, medications and procedures, levels of oxidative stress, and migration history, due to the lack of relevant information in the database. Third, although we averaged measurements for the left and right hands and then divided the participants into quartile groups, this representative value may not be the most appropriate measure of handgrip strength for each participant. Lastly, considering the homogenous population of the current study, the outcomes identified in the association of handgrip strength and cognitive function from this study may not entirely imply to all cultural concepts. As addressed above, the gender effects and age differences identified may have resulted from a Korean or more broadly East Asian cultural perspective [40,41,42]. Given that a previous study reported a significant difference in handgrip strength among races [54], further research is required with more diverse and heterogenous population, being inclusive of various other cultures, investigating this association. Additionally, to compensate the absence of confounding factors of the current study, future research should consider incorporating EEG patterns, intelligence quotients, and detailed cognitive function tests to evaluate the association between muscle strength and cognitive decline in more depth.

## 5. Conclusions

In conclusion, the present study demonstrated a significant positive association between baseline handgrip strength and later cognitive function in older adults according to sex. Considering the importance of monitoring and treating cognitive impairments, timely assessment of lower handgrip strength may be useful for the early identification of older adults at risk for cognitive impairment, especially for females as they may be at a greater risk than males. Further studies are required to elucidate the mechanisms underlying this association to aid the development of appropriate intervention strategies to prevent decreases in cognitive function according to sex and early levels of handgrip strength.

## Figures and Tables

**Table 1:** Handgrip strength and K-MMSE scores according to sex at baseline.

Variables	Males (N = 989)			Females (N = 761)			*p*
	Mean	SE	95% CI	Mean	SE	95% CI	
Handgrip strength	29.38	0.18	29.02–29.74	18.09	0.15	17.81–18.38	<0.001
K-MMSE	27.49	0.06	27.37–27.61	26.87	0.07	26.73–27.01	<0.001

K-MMSE: Korean Mini-Mental State Examination; SE: standard error; CI: confidence interval.

**Table 2:** Baseline characteristics of the study participants according to sex.

Variables		Males	Females	*p*
Handgrip strength	Handgrip 1 (strongest)	249 (25.2)	202 (26.5)	0.176
	Handgrip 2	271 (27.4)	181 (23.8)	
	Handgrip 3	224 (22.6)	199 (26.2)	
	Handgrip 4 (weakest)	245 (24.8)	179 (23.5)	
Age (years)	65–69	466 (47.1)	220 (51.4)	0.079
	70–74	297 (30.0)	220 (28.9)	
	75–79	161 (16.3)	119 (15.6)	
	≥80	65 (6.6)	31 (4.1)	
Educational attainment	≤Elementary school	435 (44.0)	565 (74.3)	<0.001
	Middle school	157 (15.9)	86 (11.3)	
	High school	263 (26.6)	93 (12.2)	
	≥College	134 (13.5)	17 (2.2)	
Economic activity	Employed	343 (34.7)	91 (12.0)	<0.001
	Unemployed	646 (65.3)	670 (88.0)	
Equalized household income	Quartile 1: low	276 (27.9)	256 (33.6)	0.039
	Quartile 2	234 (23.6)	169 (22.2)	
	Quartile 3	233 (23.6)	148 (19.5)	
	Quartile 4: high	246 (24.9)	188 (24.7)	
Marital status	Married	910 (92.0)	423 (55.6)	<0.001
	Unmarried (single, divorced, widowed)	79 (8.0)	338 (44.4)	
Residential area	Urban	623 (63.0)	519 (68.2)	0.023
	Rural	366 (37.0)	242 (31.8)	
Alcohol consumption	Never	284 (28.7)	656 (86.2)	<0.001
	Former drinker	165 (16.7)	17 (2.2)	
	Current drinker	540 (54.6)	88 (11.6)	
Smoking	Never	420 (42.5)	729 (95.8)	<0.001
	Former smoker	261 (26.4)	6 (0.8)	
	Current smoker	308 (31.1)	26 (3.4)	
Physical activity	Active	440 (44.5)	290 (38.1)	0.007
	Inactive	549 (55.5)	471 (61.9)	
Chronic diseases	No	501 (50.7)	359 (47.2)	0.037
	With one chronic disease	356 (36.0)	267 (35.1)	
	With two or more chronic diseases	1323 (13.3)	135 (17.7)	
Depression	No	935 (94.5)	687 (90.3)	<0.001
	Yes	54 (5.5)	74 (9.7)	
Body mass index (BMI)	Underweight	44 (4.5)	36 (4.7)	<0.001
	Normal weight	474 (47.9)	301 (39.6)	
	Overweight	285 (28.8)	224 (29.4)	
	Obesity	(18.8)	200 (26.3)	

Values are presented as numbers (%). *p*-values were determined using weight-adjusted chi-square tests.

**Table 3:** Multivariable analysis of factors associated with K-MMSE scores for males and females.

Variables	Males				Females			
	Unadjusted ^†^		Adjusted ^‡^		Unadjusted ^†^		Adjusted ^‡^	
	β	SE	β	SE	β	SE	β	SE
Handgrip strength								
Group 1 (strongest)	Ref		Ref		Ref		Ref	
Group 2	−0.35 *	0.15	−0.21	0.15	−0.32	0.19	−0.20	0.18
Group 3	−0.51 **	0.16	−0.28	0.16	−0.79 ***	0.19	−0.65 ***	0.19
Group 4 (weakest)	−1.04 ***	0.16	−0.54 ***	0.16	−0.78 ***	0.19	−0.53 **	0.19
Age (years)								
65–69			Ref.				Ref.	
70–74			−0.01	0.11			0.10	0.13
75–79			−0.11	0.14			0.03	0.17
≥80			−0.63 ***	0.19			−0.68 **	0.25
Survey year (wave)								
1st: 2006			Ref.				Ref.	
2nd: 2008			−1.35 ***	0.12			−2.06 ***	0.16
3rd: 2010			−1.69 ***	0.15			−2.50 ***	0.20
4th: 2012			−1.84 ***	0.17			−2.52 ***	0.22
5th: 2014			−2.41 ***	0.20			−3.54 ***	0.24
6th: 2016			−2.86 ***	0.23			−3.72 ***	0.27
7th: 2018			−3.89 ***	0.28			−4.63 ***	0.31
Educational attainment								
≤Elementary school			Ref.				Ref.	
Middle school			0.40 *	0.16			0.54 *	0.21
High school			0.78 ***	0.14			0.92 ***	0.21
≥College			1.02 ***	0.18			1.62 ***	0.45
Economic activity								
Unemployed			Ref.				Ref.	
Employed			0.49 ***	0.11			0.26	0.17
Equalized household income								
Quartile 1: low			Ref.				Ref.	
Quartile 2			0.20	0.12			0.09	0.14
Quartile 3			0.34 **	0.13			0.09	0.15
Quartile 4: high			0.03	0.13			−0.09	0.15
Marital status								
Married			Ref.				Ref.	
Unmarried (single, divorced, widowed)			0.28	0.18			−0.07	0.13
Residential area								
Urban			Ref.				Ref.	
Rural			−0.41 ***	0.12			−0.58 ***	0.14
Alcohol consumption								
Never			Ref.				Ref.	
Former drinker			−0.34 *	0.15			−0.68 *	0.34
Current drinker			−0.03	0.12			−0.05	0.20
Smoking								
Never			Ref.				Ref.	
Former smoker			−0.16	0.13			−0.24	0.57
Current smoker			0.07	0.13			0.55	0.36
Physical activity								
Inactive			Ref.				Ref.	
Active			0.35 ***	0.09			0.48 ***	0.11
Chronic diseases								
No			Ref.				Ref.	
with one chronic disease			−0.03	0.11			0.03	0.14
with two or more chronic diseases			−0.36 *	0.15			−0.07	0.17
Depressive symptoms								
No			Ref.				Ref.	
Yes			−1.48 ***	0.15			−1.01 ***	0.15
Body mass index (BMI)								
Underweight			0.19	0.20			−1.01 ***	0.24
Normal weight			Ref.				Ref.	
Overweight			0.28 **	0.11			0.27 *	0.13
Obesity			0.01	0.14			0.24	0.15

K-MMSE: Korean Mini-Mental State Examination. Values are presented as estimates (β) and standard errors (SE). * *p* < 0.05, ** *p* < 0.01, *** *p* < 0.001. ^†^ Unadjusted: estimates (β) for handgrip strength. ^‡^ Adjusted: estimates (β) were adjusted for handgrip strength, age, survey year, socioeconomic variables including educational attainment, economic activity, household income, marital status, and residential area; and health-related variables including alcohol consumption, smoking, physical activity, chronic diseases, depressive symptoms, and body mass index.

## Data Availability

The KLoSA is a biennial survey of nationally representative Koreans aged over 45 years, and the de-identified data are publicly available from the Korea Employment Information Service website (http://survey.keis.or.kr, accessed on 10 November 2021).

## Supplementary Materials

The following are available online at https://www.mdpi.com/article/10.3390/ijerph19031048/s1, Figure S1: Changes in mean K-MMSE scores of males according to handgrip strength groups during the seven waves of KLoSA. K-MMSE: Korean Mini-Mental State Examination, KLoSA: Korean Longitudinal Study of Aging, Figure S2: Changes in mean K-MMSE scores of females according to handgrip strength groups during the seven waves of KLoSA. K-MMSE: Korean Mini-Mental State Examination, KLoSA: Korean Longitudinal Study of Aging.
