# Supplementary material for: Association between Handgrip Strength and Cognitive Function in Older Adults: Korean Longitudinal Study of Aging (2006–2018)
Source: Int J Environ Res Public Health. 2022 Jan 18;19(3):1048. doi: 10.3390/ijerph19031048 (PMC8833993; doi:10.3390/ijerph19031048)
Supplement: Supplementary file 1 [file ijerph-19-01048-s001.zip › ijerph-1510021-supplementary.pdf]

Appendix

Association between handgrip strength and cognitive function in older adults: Korean Longitudinal Study of Aging (2006–2018)

Corresponding author: wchung@yuhs.ac

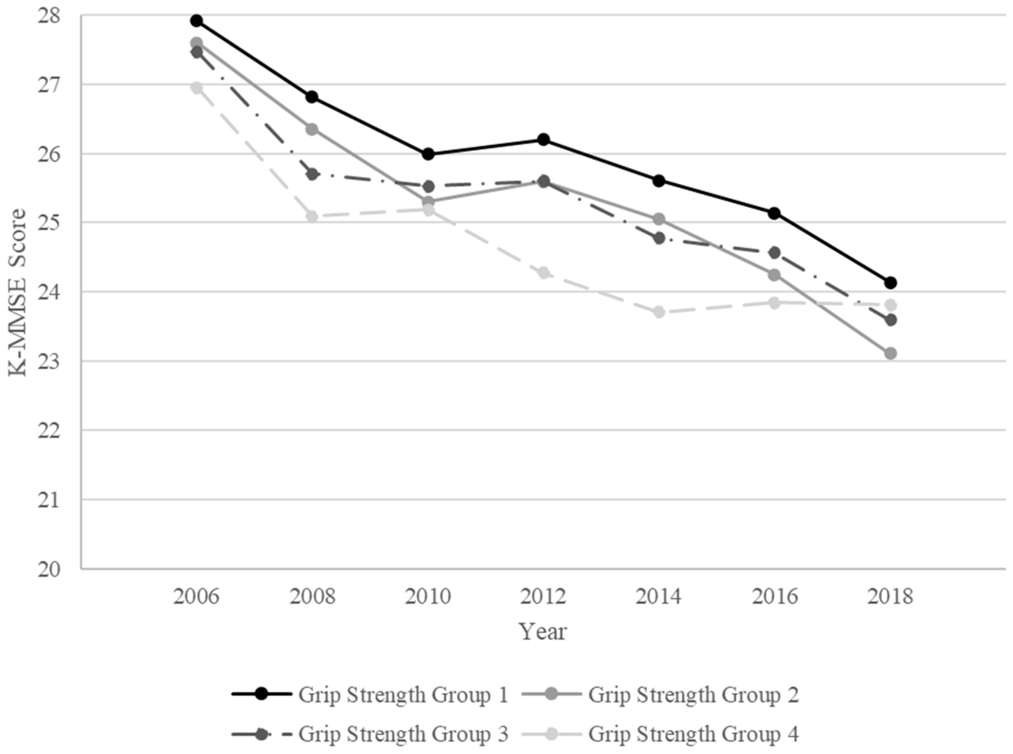

Supplementary Figure S1. Changes in mean K-MMSE scores of males according to handgrip strength groups during the seven waves of KLoSA. K-MMSE: Korean Mini-Mental State Examination, KLoSA: Korean Longitudinal Study of Aging.

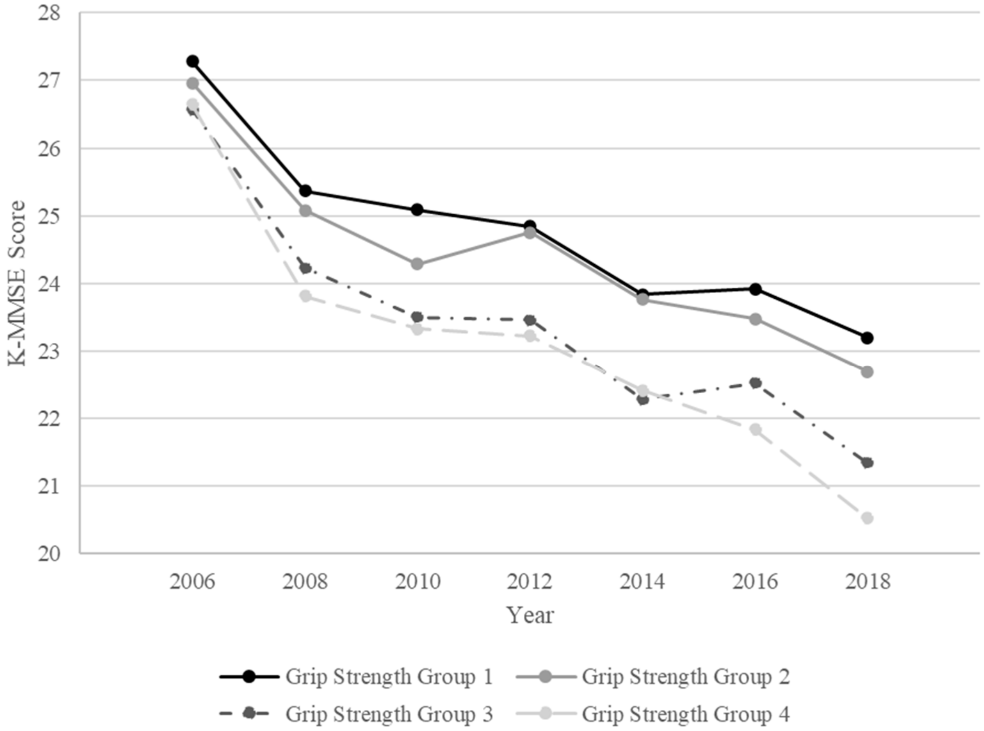

Supplementary Figure S2. Changes in mean K-MMSE scores of females according to handgrip strength groups during the seven waves of KLoSA. K-MMSE: Korean Mini-Mental State Examination, KLoSA: Korean Longitudinal Study of Aging.
